# Association between Alcohol Consumption, Folate Intake, and Risk of Pancreatic Cancer: A Case-Control Study

**DOI:** 10.3390/nu9050448

**Published:** 2017-05-01

**Authors:** Winta Yallew, William R. Bamlet, Ann L. Oberg, Kristin E. Anderson, Janet E. Olson, Rashmi Sinha, Gloria M. Petersen, Rachael Z. Stolzenberg-Solomon, Rick J. Jansen

**Affiliations:** 1 Department of Public Health, North Dakota State University, Fargo, ND 58102, USA; winta.yallew@ndsu.edu; 2 Department of Health Sciences Research, Mayo Clinic, Rochester, MN 55905, USA; Bamlet.William4@mayo.edu (W.R.B.); Oberg.Ann@mayo.edu (A.L.O.); Olson.Janet@mayo.edu (J.E.O.); Petersen.Gloria@mayo.edu (G.M.P.); 3 Department of Epidemiology and Community Health, University of Minnesota, Minneapolis, MN 55455, USA; ander116@umn.edu; 4 Division of Cancer Epidemiology and Genetics, National Cancer Institute, Bethesda, MD 20850, USA; sinhar@mail.nih.gov; 5 Department of Epidemiology, National Institutes of Health, Bethesda, MD 20850, USA; Rachael.Solomon@nih.gov

**Keywords:** pancreatic cancer, alcohol intake, folate intake, case-control study

## Abstract

Pancreatic cancer is one of the most fatal common cancers affecting both men and women, representing about 3% of all new cancer cases in the United States. In this study, we aimed to investigate the association of pancreatic cancer risk with alcohol consumption as well as folate intake. We performed a case-control study of 384 patients diagnosed with pancreatic cancer from May 2004 to December 2009 and 983 primary care healthy controls in a largely white population (>96%). Our findings showed no significant association between risk of pancreatic cancer and either overall alcohol consumption or type of alcohol consumed (drinks/day). Our study showed dietary folate intake had a modest effect size, but was significantly inversely associated with pancreatic cancer (odds ratio (OR) = 0.99, *p* < 0.0001). The current study supports the hypothesis that pancreatic cancer risk is reduced with higher food-based folate intake.

## 1. Introduction

Pancreatic cancer is one of the most fatal common cancers affecting both men and women, and represents about 3% of all new cancer cases in the United States (US) [1]. Pancreatic cancer is associated with later age of onset, with the median age at diagnosis being 71 years [1]. In the US for 2016, the estimated incidence is nearly 53,070 with a mortality rate of 79% [1]. Although pancreatic cancer is relatively rare, it has the highest fatality rate (90%) among cancers, with a less than 10% five year survival rate in the US [2].

Lack of screening tests, limited knowledge about the cause of pancreatic cancer, and delayed onset of symptoms of the disease tend to contribute to the low survival rate of the cancer [2]. Various studies have indicated that family history of pancreatic cancer, obesity, diabetes, inflammation of the pancreas, alcohol use, and cigarette smoking are associated with the risk of developing pancreatic cancer [3,4].

Numerous studies have examined the association between alcohol consumption and pancreatic cancer risk, but results are inconsistent [5]. Whereas some studies concluded that an increased risk of pancreatic cancer is associated with heavy alcohol drinking, other studies indicated a lack of association between alcohol intake and the risk of pancreatic cancer [6]. In a pooled analysis of 14 cohort studies, excess risk of pancreatic cancer was observed among individuals consuming 30 or more grams of alcohol per day [7]. A meta-analysis of prospective cohort and case-control studies reported an increased risk of pancreatic cancer with alcohol intake of nine or more drinks per day [6]. Additionally, multiple cohort studies have found no association between alcohol consumption and pancreatic cancer [8,9,10,11]. Part of the difficulty in comparing individual studies from different areas of the world is the wide variation in types of beverage consumed and varied alcohol concentration even within one alcoholic beverage type.

Folate is one of the essential vitamins in foods such as beans, lentils, and spinach, and is also available as a dietary supplement [12]. Existing evidence indicates the important role of folate in DNA methylation, synthesis and repair that subsequently reduces the risk of developing cancer [13]. Numerous epidemiologic studies have shown that folate is protective against colorectal, gastric, and other cancers. However, epidemiologic studies examining the relationship between folate intake and risk of pancreatic cancer have been limited [2]. A prospective cohort study reported a significant correlation of high dietary folate intake with reduced risk of pancreatic cancer [14]. Higher consumption of daily folate compared with low folate intake (<200 μg/day) showed a pancreatic cancer risk reduction for dietary folate (OR = 0.25, 95% confidence interval (CI) = 0.11–0.59; *p* = 0.002) and for total folate (supplemental and dietary) (OR = 0.33, CI = 0.15–0.72; *p* = 0.01) [14]. However, an analysis of 14 prospective cohort studies (2195 cases out of 862,664 people) from different countries indicated a null association with either dietary (risk ratio (RR) = 1.06, CI = 0.90–1.25, *p* = 0.47) or total folate (supplemental and dietary) (RR = 0.96, CI = 0.80–1.16, *p* = 0.90) intake and risk of pancreatic cancer [15]. In the Alpha-Tocopherol, Beta-Carotene Cancer Prevention Study cohort, 157 out of 27,101 healthy male smokers aged 50–69 years developed pancreatic cancer, and the adjusted hazard ratio comparing the highest with the lowest quintile of dietary folate intake was 0.52 (95% confidence interval: 0.31, 0.87; *p*-trend = 0.05) [16]. In the European Prospective Investigation into Cancer and Nutrition (EPIC), a nested case-control study was conducted to investigate potential biomarkers of folate intake [17]. Among 463 cases and matched controls, using plasma folate categories of ≤5, 5–10, 10–15 (reference), 15–20, and >20 nmol/L resulted in ORs of 1.58 (95% CI = 0.72–3.46), 1.39 (0.93–2.08), 1.0 (reference), 0.79 (0.52–1.21), and 1.34 (0.89–2.02), respectively [17].

Folate is one of the essential vitamins in foods such as beans, lentils, and spinach, and is also available as a dietary supplement [12]. Existing evidence indicates the important role of folate in DNA methylation, synthesis and repair that subsequently reduces the risk of developing cancer [13]. Numerous epidemiologic studies have shown that folate is protective against colorectal, gastric, and other cancers. However, epidemiologic studies examining the relationship between folate intake and risk of pancreatic cancer have been limited [2]. A prospective cohort study reported a significant correlation of high dietary folate intake with reduced risk of pancreatic cancer [14]. Higher consumption of daily folate compared with low folate intake (<200 μg/day) showed a pancreatic cancer risk reduction for dietary folate (OR = 0.25, 95% confidence interval (CI) = 0.11–0.59; *p* = 0.002) and for total folate (supplemental and dietary) (OR = 0.33, CI = 0.15–0.72; *p* = 0.01) [14]. However, an analysis of 14 prospective cohort studies (2195 cases out of 862,664 people) from different countries indicated a null association with either dietary (risk ratio (RR) = 1.06, CI = 0.90–1.25, *p* = 0.47) or total folate (supplemental and dietary) (RR = 0.96, CI = 0.80–1.16, *p* = 0.90) intake and risk of pancreatic cancer [15]. In the Alpha-Tocopherol, Beta-Carotene Cancer Prevention Study cohort, 157 out of 27,101 healthy male smokers aged 50–69 years developed pancreatic cancer, and the adjusted hazard ratio comparing the highest with the lowest quintile of dietary folate intake was 0.52 (95% confidence interval: 0.31, 0.87; *p*-trend = 0.05) [16]. In the European Prospective Investigation into Cancer and Nutrition (EPIC), a nested case-control study was conducted to investigate potential biomarkers of folate intake [17]. Among 463 cases and matched controls, using plasma folate categories of ≤5, 5–10, 10–15 (reference), 15–20, and >20 nmol/L resulted in ORs of 1.58 (95% CI = 0.72–3.46), 1.39 (0.93–2.08), 1.0 (reference), 0.79 (0.52–1.21), and 1.34 (0.89–2.02), respectively [17].

Several researchers have studied the metabolic interaction of alcohol consumption and folic acid. Based on studies done on primates, alcohol consumption is associated with reduced activity of two proteins (glutamate carboxypeptidase (GCPII) and reduced folate carrier (RFC)) that regulate folate intestinal absorption, decreased liver uptake, and accelerated renal excretion of folic acid that ultimately result in folate deficiency [18]. Similarly, Mason et al. [19] also demonstrated that alcohol consumption reduces the concentration of dietary folate through inhibition of folate-mediated methionine synthesis, which subsequently hinders the methylation process and increases the risk of developing cancer.

Thus, while researchers have examined either the relationship between pancreatic cancer and alcohol consumption or folate intake, the correlation between these dietary components and the risk of pancreatic cancer remains inconsistent. Therefore, the objective of this study was to more definitively investigate the associations between alcohol or folate intake and pancreatic cancer risk. 

## 2. Materials and Methods 

The participant recruitment strategy and data collection process have been detailed elsewhere [20,21]. Briefly, a total of 2473 patients, diagnosed with pancreatic cancer from May 2004 to December 2009, were recruited from Mayo Clinic during their clinical evaluation. Of the 2473 pancreatic cases identified, 67% (*n* = 1648) agreed to participate in this study. More than 80% of cases were confirmed based on histologic findings and the remainder were confirmed using clinical criteria. A total of 2708 potential controls with similar demographic characteristics but without pancreatic cancer were recruited from the primary care clinics of Mayo Clinic within the same time frame. Of the 2708 eligible controls identified, 1514 agreed to be part of this study. Controls were frequency matched to cases on age during enrollment, race, sex, and address. Subjects with prior history of pancreatitis and other cancers were excluded from the control pool.

The study protocol was reviewed and approved by the Mayo Clinic Institutional Review Board. All eligible individuals provided written informed consent to participate in the study. Information on demographic characteristics, dietary intake, body mass index (BMI), alcohol consumption, folate intake, lifestyle, comorbid conditions, family history of pancreatic cancer and smoking habits were collected using a self-administered questionnaire for both cases and controls. All participants were asked to answer questions about average dietary intake, alcohol consumption and smoking status in reference to the five-year period prior to study entry (for pancreatic cancer cases, intake during this period prior to experiencing symptoms). Overall, 1397 surveys were returned by cases and controls combined; 30 surveys were incomplete and excluded from the study. Our final study population included 384 cases and 983 controls. Demographic comparisons between participants who returned or did not return the Food Frequency Questionnaire have been described elsewhere [20].

Dietary intake history included the average intake and frequency of vegetables, high fiber diets, fruits, supplemental folate consumption and meats per day. Information with regard to alcohol consumption included the number of drinks per day for each type of alcoholic drinks and the number of days per week [5]. In addition to the information on alcohol consumption, participants were also asked about specific types of alcoholic beverage intake (beer, wine and liquor) and compared to nondrinkers of each type, respectively. Information on folate intake was calculated based on the self-reported consumption of foods and vitamins using the National Institutes of Health (NIH) Diet History Questionnaire and diet *Calc formulations. Folate variables were investigated as total folate (supplemental and dietary), natural folate (folate from foods), supplemental folate (folate and folic acid from vitamins), and synthetic folate (folic acid from fortified foods and supplements).

Data analyses were performed using SAS ^®^ 9.4 software [22], and the statistical significance level was set at *p* = 0.05. Distributions of demographic patterns were assessed using frequency tables and potential risk factors were computed using logistic regression. Odds ratios (OR) and 95% confidence intervals (CI) were calculated to estimate the association between alcohol consumption, folate intake, and pancreatic cancer risk. For all models, we adjusted for other pancreatic cancer risk factors: age, sex, body mass index (BMI; <18, 18–<25, 25–<30, 30+), diabetes mellitus type 2 (DM; No, DM onset <3 years ago, DM onset >3 years ago), smoking status (Never, Current, Former quit <10 years ago, Former quit >10 years ago), and total energy intake. The alcohol-specific models included the additional adjustment factor of total folate (mcg), and the folate-specific models included the additional adjustment factor of total alcohol (g).

## 3. Results

The distributions of study participant demographic characteristics are presented in Table 1. Cases were more likely to be men (58%) and less likely to be never smokers. The participant mean age was similar for both cases and controls. Study participants were predominantly white (98%). Most baseline characteristics did not differ noticeably between pancreatic cancer cases and controls. However, DM with less than three years onset and BMI > 30 were higher among cases than controls. The majority of enrolled participants (56%) reported alcohol consumption of less than one drink per day. The average daily folate consumption was slightly higher among controls than cases. The cases and controls with low folate intake had similar characteristics to the cases and controls overall (Supplemental Table S1), but were more likely to drink less and have a higher BMI.

Table 2 shows the odds ratio (OR) estimates for alcohol consumption presented by drinks per week and by alcohol beverage type. Overall adjusted OR presented in drinks per weeks showed no significant association between alcohol intake and the risk of pancreatic cancer. OR estimates across participants who consume excess alcohol (>9 drinks/week) compared to 0–<1 drinks/week were almost the same as those who consume 0 compared to 0–<1 drinks/week, with no evidence of increased risk for pancreatic cancer. OR estimates by type of alcohol (drinks/day) were also not significant, however, those who reported not drinking any wine were suggested to be at an increased risk compared to those who reported consuming 0–<1 drinks/day (OR = 1.32; 95% CI 0.94, 1.86; *p* < 0.34). 

The relationship between folate intake and risk of pancreatic cancer is summarized in Table 3. Our study found natural (dietary) folate intake (mcg) was inversely associated with pancreatic cancer (OR = 0.99; 95% CI 0.99, 0.99; *p* < 0.0001). No significant association of pancreatic cancer with either synthetic (OR = 1.00; CI 1.00, 1.00; *p* = 0.56) or supplemental folic acid (OR = 1.00; CI 0.99, 1.00; *p* = 0.44) consumption was shown. Higher consumption of natural folate (≥267.66 mcg) compared to lower intake (<188 mcg) showed a significant decreased risk of pancreatic cancer (OR = 0.41; CI 0.25, 0.68; *p* = 0.013). 

## 4. Discussion

In this case-control study, we found no significant association between alcohol consumption and the risk of pancreatic cancer. Our findings are consistent with some previous studies [6,10,11] that have reported a null relationship between alcohol consumption and the risk of pancreatic cancer. Moreover, compared to non-drinkers, the ORs for pancreatic cancer were similar for light drinkers (between 1–3 drinks/week) and participants who consumed 3–9 drinks/week. Although numerous studies [7,9,23,24], which include large cohort and pooled study designs, have indicated an increased risk of pancreatic cancer with heavy alcohol consumption, the current study found no significant trends in risk of pancreatic cancer with increasing frequency of alcohol intake. A lack of association in our and other studies is likely due to a limited sample size and potential residual confounding by smoking.

On the other hand, studies have indicated a strong association between alcohol types and increased risk of pancreatic cancer [13,14]. This study, however, did not observe significant associations between the different types of alcohol consumption and risk pancreatic cancer. We observed a non-significant, but suggestive increased risk of pancreatic cancer associated with nondrinkers of wine compared to those who reported drinking 0–<1 drinks/day. Although speculative, it is possible that the nondrinkers group may be former drinkers who stopped drinking for health reasons or symptoms of disease. Such associations have been observed in other studies, including cohort studies [7,11].

The lack of excess risk with heavy drinking in our study may be attributed to several factors. Given the high proportion of light drinkers and very low heavy drinkers in our study, information on the amount of alcohol consumed may not be reliable. Our study sample size is relatively small, so that an association for heavy alcohol consumption, especially when stratified by type of alcohol, could not be discerned. Furthermore, since consumption of large amounts of alcohol may be prone to social marginalization, respondents may purposely or unintentionally underreport heavy drinking which could make our study more vulnerable to misclassification and reduce the power to detect differences between the groups. 

Our study highlighted an inverse relationship between natural (food-based, but not from supplements) folate intake and the risk of pancreatic cancer. Moreover, we observed a statistically significant excess risk of pancreatic cancer risk among participants with low dietary folate intake compared with those who consume the highest amounts of dietary folate (≥267.66 mcg). Similar to our findings, Larsson and colleagues [14] indicated a significant reduction of pancreatic cancer risk with higher intake of dietary folate compared to low dietary folate consumption. Foods high in folate or folic acid include: beef liver, spinach, black-eyed peas, breakfast cereals, rice and asparagus. Per serving and depending on the specific food, they provide between 89 and 215 mcg [25].

Although both of our cases and controls are selected from the same institution, recruiting our controls from a different department of the hospital might increase the likelihood of sampling bias. Misclassification bias is likely to have occurred to some extent because of the use of a self-administered food-frequency questionnaire to assess dietary intake or because of the social stigma attached to higher levels of alcohol intake. Additionally, as with most case-control studies, participants may have more difficulty recalling the past five-year exposure history. Recall bias for this and other self-reported data is one of the major limitations of this study. We analyzed and present folate variables in units of mcg/day. Folate has lower bioavailability than folic acid, so when mechanistic-based endpoints (rather than relative estimates as used here) are considered, dietary equivalents should be used (1 mcg food folate ≈ 0.6 mcg folic acid from fortified foods or supplements). Using folate equivalents of mcg/day for our total folate variable as an adjustment in the alcohol analysis does not result in any different interpretations of the data.

## 5. Conclusions

The findings in this study do not support an association between alcohol consumption and risk of pancreatic cancer or a relationship among different types of alcoholic beverage consumed and risk of pancreatic cancer. This study supports the protective effect of higher dietary folate intake against the risk of developing pancreatic cancer. Further research is necessary to explore the detailed interaction and pathophysiological changes that result in pancreatic cancer and how these dietary components may play a role in that process.

## Figures and Tables

**Table 1:** Demographic characteristics of 384 cases and 983 controls.

Variable Name	Cases (*N* = 384)		Controls (*N* = 983)	
	*N*	%	*N*	%
Gender					
Female	163	42.5	500	50.9
Male	221	57.6	483	49.1
Race					
American Indian/Alaskan Native	0	0	4	0.4
Asian/Asian-American	3	0.8	8	0.8
Black/African American	4	1.0	1	0.1
White/Caucasian	373	97.1	966	98.3
Multiracial	4	1.0	4	0.4
Alcohol (drinks/day) *					
0	81	21.1	177	18.0
0–<1	207	53.9	562	57.2
1–<3	72	18.8	184	18.7
3+	24	6.3	60	6.1
Alcohol (drinks/week) *					
0	81	21.1	177	18.0
0–<1	69	18.0	180	18.3
1–<3	77	20.1	214	21.8
3–<9	74	19.3	204	20.8
9+	83	21.6	208	21.2
Smoking Status					
Never	160	41.9	539	55.1
Former	163	42.7	402	41.1
Current	59	15.5	37	3.8
Diabetes (DM) Status					
Non-DM	229	59.6	919	93.5
DM, onset < 3 years ago	35	9.1	43	4.4
DM, onset 3+ years ago	120	31.3	21	2.1
Body Mass Index (BMI) Status kg/m^2^					
<18.5	5	1.3	5	0.5
18.5–<25	119	31.4	356	37.3
25–<30	154	40.6	422	44.2
30+	101	26.7	171	17.9
	Mean	SD	Mean	SD
Age	67.59	10.5	66.35	10.9
Total Folate (mcg/day)	651.53	303.1	711.45	310.6

Abbreviations: *N* = number, % = percent, and SD = standard deviation, DM = Diabetes Mellitus, BMI = Body Mass Index. * One drink was defined as 12 ounces of beer, 5 ounces of wine, or 1.5 ounces of 80 proof liquor, all equal to 13–14 g of alcohol.

**Table 2:** Adjusted odds ratio (OR) for alcohol consumption and alcohol type.

Variable	Case/Control (*N*)	OR ^	Lower 95% CI	Upper 95% CI	*p*
Alcohol (g)	384/983	1.00	0.99	1.01	0.883
Alcohol consumption (drinks/week) *					
0	81/177	1.10	0.70	1.73	0.538
0–<1	69/180	1.00 (referent)			
1–<3	77/214	1.00	0.64	1.57	0.962
3–<9	74/204	1.00	0.63	1.59	0.973
9+	83/208	0.94	0.59	1.49	0.624
Alcohol type (drinks/day) *					
***Beer***					
0	163/396	1.05	0.75	1.49	0.407
0–<1	199/533	1.00 (referent)			
1+	22/54	0.77	0.40	1.47	0.378
***Wine***					
0	159/290	1.32	0.94	1.86	0.335
0–<1	197/604	1.00 (referent)			
1+	28/89	1.19	0.72	1.97	0.890
***Liquor***					
0	170/390	1.02	0.72	1.42	0.670
0–<1	178/509	1.00 (referent)			
1+	36/84	0.87	0.52	1.47	0.582

Abbreviations: CI = Confidence Interval. ^ Adjusted for age, sex, smoking categories (Never, Former, Current), usual BMI (<18.5, 18.5–<25, 25–<30, 30+), diabetes categories (No, onset < 3, onset ≥ 3), total folate (mcg/day), total energy intake (quantiles), and total drinks per day of the other types of alcohol (not used with total alcohol variables). * One drink was defined as 12 ounces of beer, 5 ounces of wine, or 1.5 ounces of 80 proof liquor, all equal to 13–14 g of alcohol.

**Table 3:** Adjusted odds ratio for folate variables ^+^.

Variable	OR ^	Lower 95% CI	Upper 95% CI	*p*
***Continuous***				
Natural folate (mcg/day)	0.99	0.99	0.99	<0.0001
Synthetic folate (mcg/day)	1.00	1.00	1.00	0.564
Supplemental folate (mcg/day)	1.00	0.99	1.00	0.436
***Categorical***				
Supplemental folate (mcg/day) *				
<400	1.00 (referent)			
400+	0.98	0.74	1.29	0.875
Natural folate (mcg/day) #				
<188.14	1.00 (referent)			
≥188.14–<267.66	0.89	0.62	1.27	0.029
≥267.66	0.41	0.25	0.68	<0.0001
Synthetic folate (mcg/day) #				
<88.87	1.00 (referent)			
≥88.87–<153.26	1.35	0.94	1.93	0.156
≥153.26	1.20	0.80	1.79	0.854

Abbreviations: CI = Confidence Interval. ^ Adjusted for age, sex, smoking categories (Never, Former, Current), usual BMI (<18.5, 18.5–<25, 25–<30, 30+), diabetes categories (No, onset < 3, onset ≥ 3), total alcohol (grams), and total energy intake (quantiles). ^+^ Folate variables were investigated as natural folate (folate from foods), supplemental folate (folate and folic acid from vitamins), and synthetic folate (folic acid from fortified foods and supplements). * Categorization based on median cutpoint. # Categorization based on tertiles.

## Supplementary Materials

The following are available online at www.mdpi.com/2072-6643/9/5/448/s1.
