# Erratum: Association between Alcohol Consumption, Folate Intake, and Risk of Pancreatic Cancer: A Case-Control Study; *Nutrients* 2017, *9*, 448

**DOI:** 10.3390/nu9070656

**Published:** 2017-06-26

**Authors:** Nutrients Editorial Office

**Affiliations:** MDPI AG, St. Alban-Anlage 66, 4052 Basel, Switzerland; nutrients@mdpi.com

Due to a mistake during the production process, there was a spelling error in one of the author names in the original published version [1]. The *Nutrients* Editorial Office therefore wishes to make these corrections to the paper:

The name of the first author “Winta Yellow” was corrected to “Winta Yallew”. The email address of the first author “winta.yallew@ndsu.edu” was corrected to “winta.yallew@gmail.com”.

We would like to apologize for any inconvenience caused to the authors and readers by this mistake. We will update the article and the original version will remain available on the article webpage.
