# Supplementary material for: Association between Alcohol Consumption, Folate Intake, and Risk of Pancreatic Cancer: A Case-Control Study
Source: Nutrients. 2017 May 1;9(5):448. doi: 10.3390/nu9050448 (PMC5452178; doi:10.3390/nu9050448)
Supplement: Supplementary file 1 [file nutrients-09-00448-s001.pdf]

Supplemental Table 1. Demographic characteristics of 146 cases and 305 controls with low folate intake (<188.14 mcg/day)

Variable Name	Cases (N=146)		Controls (N=305)	
	N*	%	N*	%
**Gender**				
Female	60	41.1	165	54.1
Male	86	58.9	140	45.9
**Alochol (drinks/day)**				
Zero	27	18.49	55	18.03
More than 0, less than 1	87	59.59	192	62.95
1-< 3	24	16.44	49	16.07
3+	8	5.48	9	2.95
**Alcohol (drinks /week)**				
0 drinks per week	27	18.49	55	18.03
< 1 drink per week	31	21.23	59	19.34
1- 3 drinks per week	33	22.6	76	24.92
3-9 drinks per week	29	19.86	71	23.28
9+ drinks per week	26	17.81	44	14.43
**Smoking Status**				
Never	56	38.62	150	49.67
Former	62	42.76	131	43.38
Current	27	18.62	21	6.95
**Diabetes Status**				
Non-DM*	87	59.59	286	93.77
DM, onset unknown	12	8.22	11	3.61
DM, onset < 3 years ago	47	32.19	8	2.62
**BMI Status**				
< 18.5	2	1.4	1	0.34
18.5-< 25	39	27.27	97	33.22
25-< 30	58	40.56	126	43.15
30+	44	30.77	68	23.29
	**Mean**	**SD***	**Mean**	**SD***
**Age**	65.96	10.91	64.17	11.23

*abbreviations: N = number, % = percent, and SD = standard deviation
DM=Diabetes Mellitus BMI=Body Mass Index
